# A 10-step guide to standardising patient-reported outcomes data collection in healthcare: insights from the health outcomes observatory (H2O) project on overcoming implementation barriers

**DOI:** 10.1186/s41687-025-00958-2

**Published:** 2025-11-28

**Authors:** Gemma Galan, Preston Long, Yolima Cossio-Gil, Francesco Patalano, Kathryn Hamilton, Anouk S. Huberts, Anouk Neureiter di Torrero, Lisa R. Otto, Alizé A. Rogge, Liselotte Fierens, Rahim Lalji, Belle H. de Rooij, Ann-Kristin Porth, Carolina E. Watson, Alexandra Kautzky-Willer, Nadia C. W. Kamminga, Tanja Stamm

**Affiliations:** 1 Health Services Research Group, Vall d’Hebron Institut de Recerca (VHIR), Vall d’Hebron Hospital Universitari, Barcelona, Spain; 2 Institute for Outcomes Research, Center for Medical Data Science, Medical University of Vienna, Vienna, Austria; 3 Vall d’Hebron Hospital Universitari, Vall d’Hebron Barcelona Hospital Campus, Passeig Vall d’, Hebron, 119-129, Barcelona 08035 Spain; 4 Novartis Pharma AG, Basel, Switzerland; 5 Florence Nightingale Faculty of Nursing and Midwifery, King’s College London, London, UK; 6 Department of Quality and Patient Care, Erasmus University Medical Center, Rotterdam, The Netherlands; 7 Center for Patient-Centered Outcomes Research, Charité – Universitätsmedizin Berlin, Corporate Member of Freie Universität Berlin and Humboldt-Universität Zu Berlin, Berlin, Germany; 8 Department of Psychosomatic Medicine, Charité – Universitätsmedizin Berlin, Corporate Member of Freie Universität Berlin and Humboldt-Universität Zu Berlin, Berlin, Germany; 9 Department of Chronic Diseases and Metabolism, KU Leuven, Leuven, Belgium; 10 Department of Research & Development, Netherlands Comprehensive Cancer Organisation (IKNL), Utrecht, The Netherlands; 11 Division of Endocrinology and Metabolism, Department of Internal Medicine III, Medical University of Vienna, Vienna, Austria; 12 Universitat Autònoma de Barcelona, Nursing Department, Faculty of Medicine, Cerdanyola Del Valles, Spain; 13 Department of Dermatology, Erasmus MC Cancer Insitute, University Medical Center, Rotterdam, The Netherlands

**Keywords:** Digital health, Health data, Europe, Implementation, Patient-reported outcomes measures, Value based health care

## Abstract

**Background:**

The use of patient-reported outcome measures (PROMs), including their electronic versions (ePROMs), is increasing in clinical practice and their value for individual patient care and health service delivery is becoming more widely recognized. However, their routine use remains limited due to the lack of structured and standardized systems for collecting and integrating this information into clinical workflows. Developing a sustainable system for ePROM data collection, requires addressing a range of organizational, technical and cultural challenges. The Health Outcome Observatory project (H2O) project aims to standardise and facilitate the concurrent collection of ePROs and clinical outcomes in routine clinical practice. This paper presents the 10-steps ePROM implementation framework to guide ePROM implementation in real-world clinical settings, based on project insights. The framework is primarily designed for implementation within individual clinical practices and hospital systems, while offering adaptable elements that can inform broader, population-level strategies.

**Methodology:**

An implementation taskforce was formed within H2O, involving academic researchers with expertise in PRO measurement, digital and data specialists, Health Care Professionals (HCP), and ePROMs implementation specialists across four countries (Austria, Germany, Netherlands and Spain) and, representing the project’s three disease areas - diabetes, inflammatory bowel disease (IBD) and cancer. Data were iteratively collected from country teams and analysed thematically to identify common challenges, solutions and implementation elements. Internal validation was conducted through member checking with country teams, feedback from Patient Advisory Board (PAB) and expert triangulation within the taskforce.

**Results:**

Ten core steps for successful ePROM implementation in clinical practice were derived. In addition, disease-specific and/or site-specific considerations such as clinical population, technology use, Information Technology (IT) literacy, demographic factors and engagement of patient and providers proves important. A significant challenge identified was establishing the required technological infrastructure and data flow needed for implementation.

**Conclusions:**

The ten steps derived provide a scalable framework that supports the integration of ePROMs into routine practice and fostering improvements in patient care by including the patient voice in a structured and useable manner. Additionally, the detailed analysis of challenges and solutions for successful ePROM implementation, based on the H2O initiative, strengthens our contribution to this field.

**Supplementary Information:**

The online version contains supplementary material available at 10.1186/s41687-025-00958-2.

## Background

Clinical outcomes such as vitals, laboratory results, and imaging findings are routinely collected in health care settings. However, these outcomes do not necessarily reflect what patients value most. Patient-reported outcomes [1] (PROs) are direct self-reports from patients about their own health and well-being, without interpretation from HCP [2]. These can be gathered through patient-reported outcome measures (PROMs) and are vital for understanding a person’s health status and disease trajectory [3, 4]. PROMs systematically record patients’ health experiences, providing standardized and measurable insights on concepts that matter most [5]. The collection and use of PRO data in routine clinical practice can enhance disease monitoring, improve communication between patient and clinician, and identify physical and psychosocial concerns [6, 7]. Consequently, the integrated use of PROs in clinical settings has been shown to improve the quality of care across various conditions [8–10]. For example, a systematic review of PROMs in oncology found strong evidence that routine collection of PROMs, with feedback enhanced patient-provider communication, increased patient satisfaction, and improved the detection of previously unrecognized problems [11].

Beyond individual benefits, standardizing the collection of PROMs enables system-level improvements. It facilitates comparisons across sites and countries, supports benchmarking and helps identify best practices. Aggregated PROM data can guide quality improvement, resource allocation, and population health monitoring [12, 13]. Without standardised approaches, variability can compromise data integrity, limit interoperability, and obscure trends that might otherwise inform evidence-based decision making at the policy or organization level. Standardization also improves the scalability of implementation efforts [14, 15].

Despite the various advantages of the inclusion of PROMs within clinical settings, implementation remains challenging [16]. Challenges to clinical practice implementation have been described to exist at the system level (i.e., cultural resistance), service level (i.e., increased cost, leadership buy-in, information technology support) and at an individual level (i.e., time requirement from HCP and scepticism of PROM data relevance) [17]. To overcome such hurdles, systematic reviews have highlighted the need to integrate various stakeholders early and co-produce a clinical practice implementation strategy [18]. Given the aforementioned implementation facilitators and barriers, it can be theorized that the integration of PROMs becomes more difficult as the complexity of a clinical practice settings increases. (i.e. increased number of clinicians/patients, more complex/diverse patient cases, larger number of services offered). Such complexity, typical of highly specialized hospital settings, may be associated with organizational barriers such as system fragmentation and high clinical workload, factors previously identified as limiting the adoption of innovations in similarly complex healthcare [19, 20].

The Health Outcomes Observatory (H2O) project is an Innovative Medicines Initiative (IMI) project which seeks to advance value-based care by integrating PROMs in large European hospital settings in Austria, Germany, Netherlands and Spain [15]. Launched in 2020, the project targets 3 disease areas (Diabetes, Cancer and Inflammatory Bowel Disease). Since its inception, H2O sought to overcome barriers to implementing PROMs in complex clinical practice settings through participatory involvement with stakeholders, defining technical and psychometric standards for PRO assessment, and through establishment of infrastructure to aid in the implementation and collection of PRO data [15].

This study aims to outline effective strategies for integrating PROM in complex clinical settings across four large European hospitals. These strategies were synthesized into a stepwise implementation model to help healthcare organizations establish standardized systems for routine PROM use. Traditionally collected on paper, PROMs are now often electronic Patient-Reported Outcome Measures (ePROMs), allowing patients to respond via web portals, mobile apps, or tablets. While the content remains the same, ePROMs offer advantages such as real-time data availability, integration with electronic health records (EHRs), automated scoring, and flexible data collection. These distinctions have significant implications for implementation, especially in routine clinical practice. Therefore, although the introduction refers broadly to PROMs, the strategies in this paper specifically focus on ePROMs.

## Methods

**Fig. 1:**
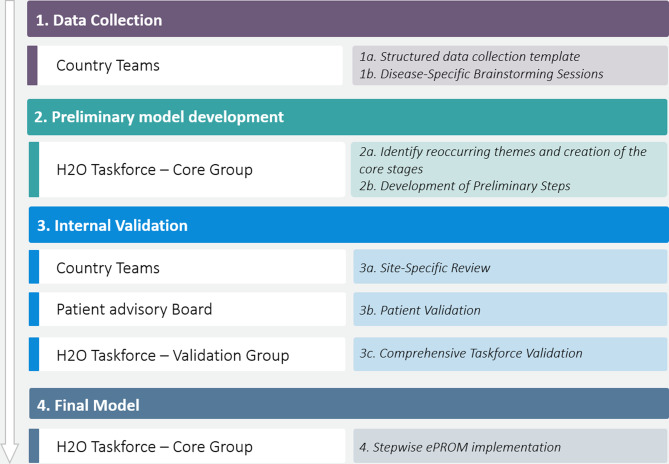
Stepwise methodology for the development of the ten steps for ePRO implementation

This study followed an iterative consensus-building approach, in which information is systematically collected, synthesized, and validated information through multiple stages. The 10-step ePROM implementation framework was developed in a multistep process (Fig. 1).

### H2O taskforce

To facilitate this process, a dedicated taskforce was established, composed of members drawn from existing working groups within the H2O project. For the purpose of this study, the taskforce was divided in two groups: A Core Group (*n* = 10) and a Validation Group (*n* = 23). The Core Group was composed of experts directly involved in the ePROMs implementation across the different participating countries. Members include academic researchers with general expertise in PRO measurement, digital and data health experts, HCP, and PROMs implementation specialists. Their primary functions included curating, analysing, verifying and synthesizing data, gathering feedback and formulating standardized implementation steps. Approximately 90% of the group were affiliated with public institutions, and 10% with private entities (see Appendix C for detailed group composition).

The Validation Group expanded the selection to include experts offering a broader, more global perspective of the project, while maintaining representation across countries and key areas of expertise (HCP, academic researchers, healthcare industry and IT specialists), reflecting the interdisciplinary nature of the H2O project. About 70% of members were affiliated with a public institution and 30% with private entities. Their main role was to review and validate the proposed model and implementation steps for feasibility and adaptability across contexts (see Appendix C for detailed group composition).

### Data collection

A structured data collection template (see Appendix A) was developed by the Core Group specifically for this study to ensure a comprehensive description of implementation plans and status. Quantitative and qualitative data were collected from the country teams via closed and open-ended questions, respectively. Country teams were formed by representatives from the local implementation sites and typically included HCP, data and digital experts, PROMs implementation specialist, and, in some cases, patient representatives and/or academic researchers (see Appendix C for detailed group composition). Specifically, data were collected on current clinical practice and patient populations, local plans, and status of H2O data collection, lessons learned from implementation, and unique implementation considerations for each country and disease area. Prior to distributing the structure data collection template, the formulated questions were discussed and agreed with the taskforce ensuring relevance, comprehension and clarity. No translations were necessary as all expected respondents (members of the country teams) were proficient in English.

The finalized structured data collection template was distributed to the country teams, who were already part of the H2O consortium, via internal project communication channels, with access provided through a shared folder link.

Data collection was conducted over two months (July-September 2023). The returned answers were supplemented with follow-up questions as needed to clarify information or request missing details via email or in-person (online). The final stage of this iterative review process, conducted in three rounds, allowed participants to revise their responses. By reviewing the feedback from their peers, participants could adjust their answers and incorporate previously unconsidered aspects, leading to a more comprehensive and holistic understanding of the implementation process. Missing data was minimal, however more often found when enquiring about implementation in cancer centres. Repeated attempts were made to ensure data completion. Data not provided was omitted from synthesis. In Table 1 an overview is provided of data elicited by the questionnaire.

**Table 1:** Overview of requested information from the questionnaire

Topic	Subtopic
Patient populations, patient pathways and current use of data	• No. patients • Proportions of Type 1 and Type 2 diabetes patients; or proportions of lung and metastatic breast cancer patients • How appointments are made • Appointment frequencies • Reminders (or other telemedical processes) • Who patients are seen by • Where any ePROM data collection fitted into the patient pathway • The clinical data collected • The PRO data collected (and PROMs used) • Reasons for collecting PRO data • How PRO data was collected • Data treatment decisions were based on • Data used to inform service improvement
Local plans for implementing the outcome sets and data collection	• Internally agreed protocols for data collection (including how ePROM data collection will fit into patient pathway) • How the ePROM data will be used • Safety follow up procedure(s) and care pathway(s) - should patients need support problems be identified via PROs respectively • Initial recruitment strategy
Current status of implementation of the outcome sets	• Maturity of the EHR • Current approach to and status of technology solutions; front- and back-end provider(s) • Actual/anticipated start date for data collection • Anticipated number of participants (n) (at start of project and per year) • Strategies for scaling up/extending recruitment to other centres. Indicated separately for: • Secondary/tertiary care Primary care • ‘Freelance’ patients (i.e. patients that will be recruited independent of an H2O centre) • Extent to which recommended outcome set is implemented in full • Strategy and timeline for achieving original proposal (including a scalable technology solution and implementation of full outcome set) • Status of ethical approval for and the start of the feasibility study
What we have learnt from implementation	• Barriers and facilitators (operating at all levels of the healthcare context) • Potential solutions to barriers and ways of harnessing learning on facilitators • Support needs (directly expressed by clinical teams)
Unique implementation considerations for each disease area	

ePROM = electronic patient-reported outcomes, PRO = patient-reported outcomes, EHR = electronic health record, H2O = health outcomes observatory

Next, brainstorming activities within disease-specific work packages were undertaken to elicit their perspective on unique implementation considerations for each disease area, center or country and lessons learned thus far.

### Preliminary Model development

The primary objective was to synthesize the information collected from country teams into an early implementation model consisting of core stages applicable across diverse context. In parallel, the analysis also served to identify relevant barriers and facilitators reported across country teams (see Appendix D). To achieve this, the Core Group conducted a comprehensive thematic analysis [21] of the collected data to identify common patterns and key elements across different disease areas and sites. Initial coding generated thematic areas that informed the preliminary implementation steps. General insights formed core themes, while context- or disease-specific findings were retained as sub-elements to preserve nuance without reducing broader applicability. This model was iteratively refined through triangulation within the Core Group, which included representatives from each participating country (see Appendix C), ensuring that the model was robust and reflective of real-world, cross-country experiences.

### Internal validation

The validation process was conducted in three phases to ensure that diverse perspectives were incorporated. First, a member-checking step was conducted with the country teams. The preliminary model was shared with them to verify that the specific contexts of their respective sites were accurately represented. Their feedback was used to refine terminology, reorder steps and clarify definitions.

Second, recognizing the importance of patient involvement, a PAB was consulted throughout this process. The PAB was composed of six patient advocates identified through patient organizations affiliated with the H2O project and selected based on their lived experience with the relevant conditions (cancer, diabetes, IBD) as well as their active involvement in patient advocacy. Collectively, they provided balanced representation across the three disease areas and came from six different European countries.

Third, a dedicated working meeting was held with the Validation Group, where taskforce members review the revised model collectively, ensuring its robustness and alignment with project objectives. Feedback was actively encouraged and minor refinements were suggested, including renaming one of the steps to better reflect its scope and improve clarity and feasibility.

After achieving agreement at this level, the ten steps were elaborated upon by selected experts within the H2O consortium. For each step, the Core Group nominated two consortium members with relevant expertise: one expert was responsible for drafting a detailed description of the step, while the other reviewed and provided feedback on the draft.

## Results

### Ten steps to PRO implementation

**Fig. 2:**
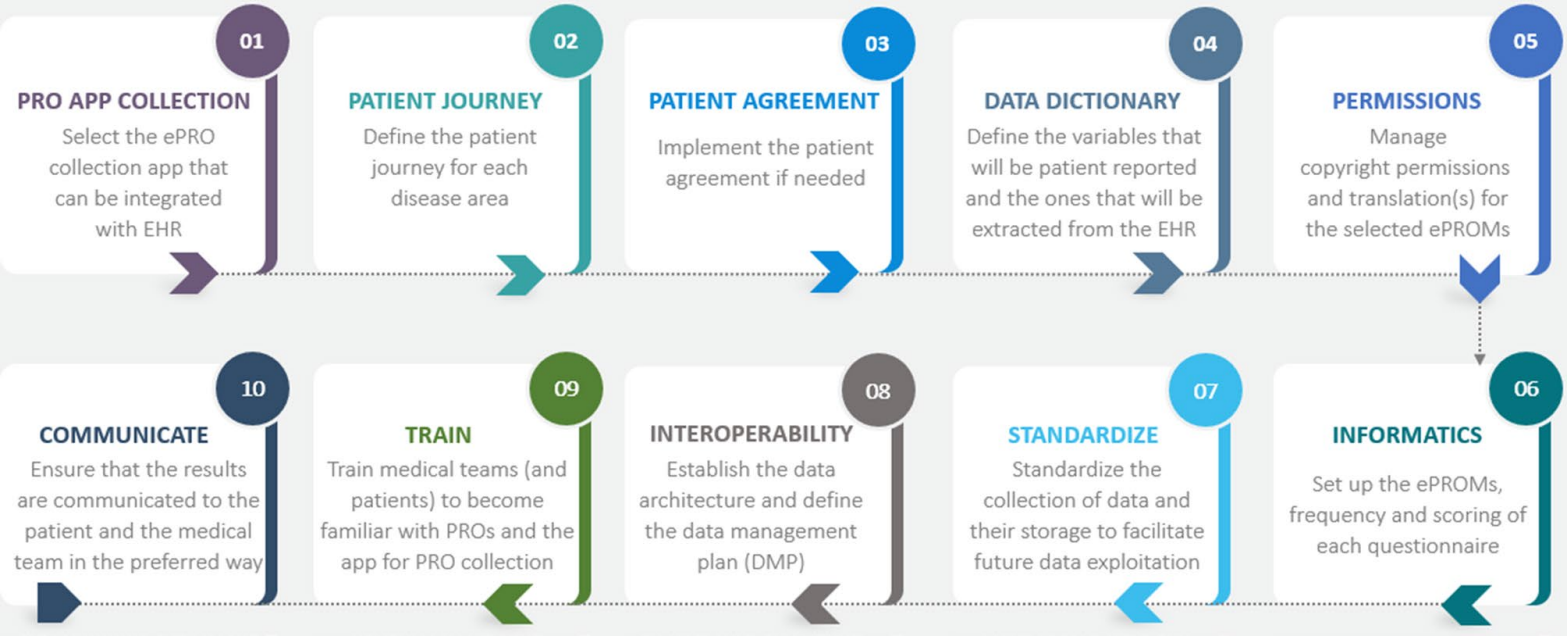
Visual overview of the ten steps identified for PRO implementation

The ten steps that were identified and formulated by the taskforce are described below, and an overview is presented in Fig. 2. The description of each step reflects the experience of implementing the data collection as specified by the H2O project and therefore, in some instances, H2O specific processes are referred to. The order of the steps may vary from one centre to another depending on their specific characteristics.

**1. Select a PRO collection app that can be integrated with the EHR.**

Integration of a PRO collection app with the EHR is key, as it complements the EHR by contributing patient-reported data. It should adhere to local governance standards, legal and technical requirements, and appropriate user features and functionalities. When considering an app (including, where appropriate, PRO functionalities available in EHR patient portals), a three-step process is recommended:

I. **Consideration of alignment** with the project’s vision and goals, including patient usability and empowerment, governance, and financial model. Actively involve patients in evaluating usability and features to ensure the app meets their needs.
II. **Conduct a technical assessment** of the app’s capabilities to securely integrate with healthcare professional-facing systems. Customisation to country and site-specific requirements is beneficial, as well as accommodating future growth and changing needs.
III. **Confirm data transfer and security requirements for final implementation.** Integration of data transfers should be finalized, and all cybersecurity and digital security measures completed.

**2. Define the patient journey for the disease area.**

Understanding the patient journey – including appointment scheduling, clinical testing and consultations – helps identify opportunities for integrating the collection and use of PRO data into clinical workflows, identifying challenges, and informing strategies to enhance patient experience. Key questions that can guide integration of PRO data collection and use into the patient journey are presented in Table 2.

**Table 2:** Questions that can guide integration of PRO data collection and use into the patient journey

Topic	Questions to ask
Access	*How can patients access the ePRO app used for data collection? Do they need to be registered? Who will explain the app to them including what is the best time to complete the ePROMs? How easy is it to use the app for ePRO collection?*
Consent	*Is the consent collected via the app enough for the planned use of the data? Does it need to be adapted to the local situation? Is it understandable for the patient?*
Existing clinical routines	*How are appointments scheduled? Where do patients arrive? Who sees the patient and when (and in which order)? How is a routine visit structured (i.e., which actions and procedures are part of a routine visit, e.g., blood draws, measurements (such as blood pressure, height, weight), consultations with nurses, educators, psychologists, etc.)?*
ePRO data collection	*When do we want to collect the ePROs (e.g. days before the clinical visit vs at the clinic shortly before the consultation)? Specifically, for the first/baseline collection point: how do people sign up (When and where?)? Will ePRO data be collected at this specific date or before the next visit? How is the timing of ePRO collection coordinated with the next scheduled appointment (i.e. how is it ensured that the patient completes the ePROMs before attending the consultation during which they will be used)? Who will explain ePROs to the patients? Who will show them how the app works? Who can provide technical support in case the patient experiences problems with the ePRO collection? Will there be dedicated (support) personnel (e.g., a specialist or nurse) or specific visits for this purpose?*
Review and communication of ePROM results	*Who is going to review the results and when? Is there a need to set up an alert-system to detect results that require (immediate) action? Who is going to communicate the results to the patient and when? How should this communication take place (i.e. which results must absolutely be discussed and what must be communicated to the patient in this regard)? What will patients be told about when their results will be seen? What will be in place to ensure that people have some access to immediate support if required/desired after completing the ePROMs? What care pathways will be in place, i.e. should problems be indicated by the ePROM data? Are there possibilities of professional support or referral to specialists (e.g. psychology, dietary advice, etc.)? And when should this be considered?*

This information facilitates refining clinical workflows – if necessary – to encompass PRO data collection and use, thereby simplifying the implementation process and ensuring strategies are tailored to both patient and clinical team needs. Actively involving patients in mapping and refining their journey is essential to identify practical considerations, improve acceptability, and optimize data collection timing. A template for disease- and centre-specific patient pathways is provided in Appendix A. This approach acknowledges potential differences in workflows even within the same hospital, across different clinical conditions. This serves as a guide that can and should be adjusted to the local context.

**3. Develop and implement a patient agreement.**

An informed consent outlining objectives, risks, and benefits of data collection, along with the process for retroactive consent withdrawal, should be established when secondary use of data is contemplated, as is the case with the H2O project. Whenever possible, it is recommended that patient consent be integrated into the data collection app, co-designed with patients when feasible and adapted to the legal framework of each participating country.

**4. Develop a data dictionary and define which variables will be patient-reported and which ones can be automatically extracted from the EHR.**

The first step is to clearly define what needs to be measured. At this stage, it is important to explore whether established guidelines, frameworks, or standardized sets of outcomes already exist for the condition or domain of interest. Useful resources include the “Consensus-based Standards for the selection of health Measurement Instruments” (COSMIN) [22] and the “International Consortium for Health Outcomes Measurement” (ICHOM) [14]. In this context, the H2O initiative has contributed significantly by developing disease-specific core outcome sets (COS) with input from key stakeholders, including patients, for clinical use [23–26] (see Appendix B). Once these outcomes are identified, a comprehensive data dictionary should be established, detailing clinical outcomes, demographic details and ePROs (including the PROMs to measure each PRO) for each health condition. This dictionary should also define the frequency and timing of data collection for each variable. Collaboration with local clinical teams and patients in this definition process is key to ensure the applicability, acceptability and feasibility of the selected indicators and timelines. Some items can be obtained directly from patients, whereas other information, depending on hospital data management and recency, can be efficiently retrieved from the EHR, reducing patient burden

**5. Manage copyright permissions and translation(s) for PROMs (or items).**

To implement PRO data collection, permission from PROM copyright holders must be obtained and all PROMs, and other items in the data dictionary should be available in the local language(s).

**A Copyright permission and translation of PROMs** To ensure appropriate and legal use of PROMs, it is important to confirm that the instrument has been validated in the intended language of use and setting. If the validity of the PROM is uncertain the criteria for defining a validated measure, the PROMIS^®^ Instrument Development and Validation – Scientific Standards [27], should be consulted. Once validity is confirmed, copyright permission for use must be sought. For some PROMs, it may be required to obtain a license for use and in some instances a fee may have to be paid. The Mapi Trust website may prove helpful with identifying copyright holders (https://eprovide.mapi-trust.org/).

When patients complete PROM questionnaires, it is essential that they do so in a language in which they feel fully comfortable and confident. Since most PROMs are originally developed in English, it is necessary to use validated translations to ensure accuracy and patient comprehension. After obtaining copyright permissions, validated translations should be identified and obtained, with citation details and permissions documented. If these cannot be sourced, copyright holders should be asked whether independent translation is allowed, and if so, whether specific translation and validation methods must be followed. Importantly, linguistic translation should be paired with cross-cultural adaptation and validation (e.g., cognitive debriefing, locale-specific review), particularly for languages used across countries. Store all translation-related correspondence and information in a translation tracker/process log.

**6. Collaborate with the medical informatics team to implement ePROMs for each disease area and set the frequency and scoring of each questionnaire.**

The data dictionaries will facilitate on-site implementation and should also include outcome categories and names; item wording, source, response options, item frequency and scoring information. Additionally, calculated fields based on PROM scoring equations should be implemented to automatically calculate total scores within the data collection infrastructure. At this point, efforts should be focused on automating the delivery of ePROMs so that the patient receives them at the appropriate times in their care journey.

**7. Standardise data collection and storage to facilitate future data use.**

It is essential to collaborate with the clinical team to determine where and how the patient-reported data will be stored in the EHR, enabling them to easily track these values. Moreover, to ensure that these data are exploitable in the future, it is crucial to consider early on how and where they will be available in earlier steps. In the H2O project, data will be stored in the OMOP Common Data Model (CDM) [28], facilitating comparisons across participating centers and countries.

**8. Interoperability: establish the data architecture or data flow and define the data management plan (DMP) as needed.**

Ensuring effective interoperability is crucial, requiring close collaboration with the IT department of healthcare centres to facilitate smooth integration with external providers or other third parties. This is particularly important when utilizing external apps for collecting ePROMs or when participating in data-sharing projects such as the H2O [15]. In the H2O project, independent entities known as National Observatories have been created to promote the integration of PROs in clinical practice and to establish a secure environment for the use of this data. In this context, a DMP detailing the processes for data collection, transfer, and sharing becomes essential, ensuring that all systems can effectively communicate while safeguarding data integrity and confidentiality. In addition, it is vital to establish and adhere to standards and protocols for data exchange, such as HL7/FHIR [29].

**9. Train medical teams and patients to become familiar with PROs and the app for ePRO collection.**

Both patients and healthcare professionals need support to become comfortable with technology used and understand the purpose and value of ePROs in routine care. HCP should be familiar with the app to effectively recommend and guide patients through its use. Patient engagement is likely to increase if they recognise the importance of PROs for the monitoring and treatment of their condition [30] and if their data is actually being used during their individual consultation and for their treatment decisions [31].

H2O offers disease area-specific information and training materials for patients and healthcare professionals to support clinical implementation [32–34]. These materials stress the importance of patient-reported information, the process of collecting and using this data, and practical tips on how to embed this into the care pathway.

Technical guidance should ideally come from the solution’s developer/owner and be available to patients and HCPs e.g., included in a frequently asked questions (FAQ) document. Training should also cover basic data privacy and information security. Technical support mechanisms should remain available for troubleshooting.

**10. Communicate results to the patient and the medical team in the preferred way.**

Communicating results effectively could not only support clinical decision-making but also help patients recognise the value of ePROs and feel that their input contributes meaningfully to their care. Efficient data collection and presentation methods should be integrated from the start. Communication between patients and healthcare teams may be enhanced through messaging functions, interoperable visuals, and clear guidance on interpreting ePRO and clinical data. Dashboards tailored for patients and healthcare professionals, should be developed collaboratively with end-users [35, 36] and tested with target populations within the clinical context to ensure they meet specific needs.

The findings reported above will be evaluated in a formal feasibility study. This ongoing study collects perceived clinical utility and user experience data throughout implementation and includes both questionnaires and interviews from HCPs and patients. A key outcome is a maturity model created for the H2O project used to indicate implementation progress across 11 relevant indicators [37].

## Discussion

This work will facilitate the implementation of ePROM collection agnostic to disease area or location. The results highlight potential pitfalls in each key step of the journey, aiding in their avoidance prior to arrival, as well as presenting solutions (see Appendix D). EHR integration, mapped patient pathways, governance/consent, standardized data dictionary, training and dashboards utilization are key to the sustained and efficient success of such an endeavor. The implementation of ePROMs in clinical practice presents several challenges that need to be addressed for successful integration. The heterogeneity of patient populations requires tailored approaches for different disease areas and patient groups. Demographic factors also play a crucial role; consideration of patient age, socioeconomic status and cultural background is essential in designing engagement strategies that ensure widespread participation and accurate data collection. This includes accurate translations made available in multiple languages to avoid excluding diverse patient groups [38]. These can be done through expert consensus and iterative forward backward translation rounds for efficiency [39, 40]. It is important to consider copyright laws at such times, and to review supportive PROM copyright guides addressing permissions, such as the ISOQOL Translation and Cultural Special Interest Group recommendations [41].

Technology use and IT literacy also vary significantly across sites and populations, necessitating solutions that can be adapted to various levels of technological proficiency. Training both patients and HCPs in using the digital solution(s), understanding PROs and their anticipated benefits, as well as their recommended use in clinical practice is crucial [30]. Training professionals can decrease resistance and shorten the time before the system generates added value [42]. The troublesome initial increase in clinician burden is one of the main hurdles that can lead to the permanent cessation of ePROM implementation, but proper training can shorten this period dramatically [43]. Training can come in the form of in-person or online sessions, newsletters, or recurring emails [44]. It is also recommended to have a champion on site who is well trained and can assist colleagues in trouble shooting [45]. Lastly, a user-friendly system may help reduce training time and increase long-term utilization.

To maximise the utility of ePROMs, and clinical outcomes, they must be quickly accessible and easy to interpret [46]. User experience testing of systems and dashboards used by patients and HCPs is essential for fostering understanding, engagement and decision making [47]. Key elements to consider include accessibility, ease of use, learnability, memorability, comprehension, and personalisation.

Integrating patient-reported input into the EHR may improve data quality [48]. The EHR may evolve into a personal health record [49], primarily filled out by patients before visits, thus offering efficiency and time-saving advantages. Integrating ePROMs into EHRs can improve patient-provider communication and delivery of care, and it is a determining factor in the routine use of PROs [50]. The significant challenge of establishing the required technological infrastructure and data flow underscores the importance of investing in robust IT systems and ensuring interoperability between different platforms.

A recent systematic literature review found that implementation checklists can be highly supportive [51]. Other examples, such as COSMIN [22] or ICHOM [14] can be essential in this process.

A stepped approach to implementation, as recommended by the H2O project, begins with a reduced (pragmatic) outcome set. This allows for gradual adaptation and refinement of methods.

A review of comparable implementation guides highlights similarities and differences. One team of researchers presents a stepwise approach that separates into three stages [17]. In this model, there is an emphasis on the research perspective, with more focus on the selection and scrutinization, and digital organization of the ePROMs. However, in our 10-steps, more emphasis is placed on the pragmatic hurdles identified such as legal, financial, and stakeholder communication considerations. Comparison with another implementation framework found a strong overlap in importance placed in HCP and patient training [52]. Frequency of collection and timing is also highlighted, however, like many other frameworks, the results presented are sourced from external literature, whereas the guide presented in this paper comes directly from firsthand accounts and simultaneous input directly from all relevant stakeholders across multiple countries and conditions, i.e. based on real-world experiences instead of purely theoretical frameworks. These alternate frameworks are often either too broadly represented to be actionable or too context-specific to be generalizable [53, 54]. Though, even detailed and actionable frameworks presented are still purely theoretical and based on reviews [13]. While we present a real-world, practical, procedural, and easy-to-follow 10-step guide to support straightforward implementation and comprehension.

The importance of initiatives like the H2O project cannot be overstated. By sharing challenges and collectively seeking solutions, these initiatives foster a community of practice that can develop and refine best practices. This collaborative approach accelerates learning and innovation, ensuring that the solutions developed are both practical and widely applicable. Moreover, joining efforts of diverse stakeholders (HCPs, IT experts, researchers, patients, etc.) create a comprehensive understanding and facilitate the development of holistic and effective solutions.

### Limitations and strengths

Our results should be interpreted in the light of several limitations. First, while this paper is a novel step-by-step guide to ePROM implementation in clinical practice, its generalizability beyond hospital settings may be limited. The ten-step approach, although comprehensive, was specifically developed from the experience of implementing ePROMs in four clinical areas: diabetes, IBD, metastatic breast cancer, and lung cancer. While the framework addresses key elements, disease-specific needs and challenges may require adaptation. This could include potential barriers in other conditions and local requirements, such as regulatory and ethical standards, which could affect the framework’s consistency across regions. Input was gathered from private industry, academics, HCP, patients, and patient representatives ensuring that all relevant viewpoints were captured. Although our expert panel did not formally include representatives from initiatives such as ICHOM, some contributors had experience collaborating with such efforts, or with related standardisation projects like EHDEN. While the resulting 10-steps model is inclusive and address gaps across the H2O consortium, the absence of external validation is a limitation. Future validation could confirm the model’s broader applicability and effectiveness. Furthermore, the inclusion of multiple sites bolsters the generalizability of our findings but also hinders the specificity that can be achieved. As a final note, we encourage all sites implementing a large-scale COS to use a stepwise approach as presented here.

Currently, H2O has included over 4,000 patients across the four disease areas. Since conception, 2 additional hospitals have been onboarded beyond the initial partners. There are 16 providers collecting the COS continuously in their centers with a current average completion rate of 80%, slightly higher than the typical 70% reported [55]. While the data dictionaries are generic in nature, i.e. intended to be used in all current and future countries, some centres have opted to collect additional outcomes or have yet to fully implement the entire COS resulting in minor variations. The first complete analysis of this data is underway, focus on patient stratification.

## Conclusions

We provide an overview of preliminary findings from the H2O project implementation process across multiple countries and disease areas and suggest a generic ten step guide for implementing integrated and standardized ePRO and clinical data collection in routine clinical care. This paper highlights the solutions identified at each implementation step while confronting challenges encountered across the disease areas and countries. The main focus areas regarded technical infrastructure and data flow, scaling up, and full implementation of recommended outcome sets. Despite challenges related to ePRO data collection and use, the potential benefits for enhancing patient care and ultimately improving the overall healthcare landscape are significant.

## Electronic supplementary material

Below is the link to the electronic supplementary material.

Supplementary Material 1

## Appendix A – Example template for data collection

**Table:**

Disease
Key players/clinical leads, key team members, etc.
**Clinical team members responses were validated by:**
**PART 1: Clinical populations, patient pathways and current use of PROs (*****prior to H2O***)
1. No. patients
2. Proportion of T1/2D patients
3. How appointments are made
4. Appointment frequencies
5. Reminders (or other telemedical processes)
6. Who patients are seen by
7. Where any ePROM data collection fitted into patient pathway
8. Clinical data collected and ePROMs used
9. Reasons for this data collection
10. How this data was collected
11. Data treatment decisions were based on
12. Data used to inform service improvement
**PART 2****: Local plans for implementing the outcome sets and data collection (*****i.e. H2O***) *(refer to local IRB applications)*
1. Internally agreed protocols for data collection (incl. how ePROM data collection will fit into patient pathway) (please provide a patient pathway flow chart)
2. How the data will be used
3. Safety follow up procedure(s). (I.e. what will be in place to ensure that people have some access to immediate support if required/desired after completing the ePROMs, e.g. advising people that scores may not be seen for some time (specifying the expected delay) and providing local contact numbers/helplines for emergencies or problems (or perhaps even listing a primary H2O contact person). The requirements for this will vary by site and depending on the approach used for the feasibility study (e.g. a study versus a registry). There will also be a requirement to specify the safety follow up procedure re: participation in the H2O ecosystem more generally (once the feasibility study is completed).
4. Care pathway(s) in place that will be followed should problems be indicated by the ePROM data
5. Initial recruitment strategy: feasibility study and H2O ecosystem
**PART 3: Current status of implementation of the outcome sets: readiness and capabilities** *(refer to original proposal)*
1. Status of EHR
2. Current approach to and status of tech solutions; front- and back-end provider(s) (incl. what has been achieved *relative to original proposal*)
3. Actual/anticipated start date for data collection *The first patient in = the first patient participating in ‘H2O’ (wherein the data is collected as part of and belongs to H2O), the first step of which is participating in the feasibility study (not the first patient to collect the COS defined by H2O, i.e. where the COS has been implemented in daily practice but not as part of H2O). Note that for some centres the first patient may come from retroactively added data.*
4. Anticipated n (at start of project and per year)
5. Strategies for scaling up/extending recruitment to other centres. Indicate for:- Secondary/tertiary care-Primary care-‘Freelance’ patients (***please complete Gantt chart re: timeline for recruitment scaling up – see template***)
6. Extent to which recommended outcome set is implemented in full
7. Local situation re: which outcomes will be patient reported and which ones will be extracted from EHR
8. Strategy and timeline for achieving original proposal (incl. a scalable tech solution and implementation of full outcome set - reality of achieving this)
9. Status of IRB approval and feasibility study
10. Ethical and informed consent requirements and approach: feasibility study and H2O ecosystem
**PART 4: Lessons learned from implementation (so far)**
*Barriers to implementation*
*Potential solutions to barriers*
*Facilitators for implementation*
*Potential ways of harnessing learning on facilitators*
*Support required*
**Context-specific considerations re: implementation**
*Disease-specific considerations re: implementation*

## Appendix B - Pragmatic COS available at link for download

**Table:**

Category	Variable
Generic Items	
Socio-demographics	Date of birth
	Sex
	Smoking status
	Alcohol use
	Weight
	Height
Case-mix variables – patient-related	Healthcare access
	Disease Diagnosis
	Year of Diagnosis
Patient Reported Outcome	Overall well-being (PROMIS − 10, 29 or 33 items)
**Diabetes Type 1 and Type 2**	
Clinical outcome	Pharmacological treatment modality
	Technological treatment modality
	HbA1c
	Mean glucose (based on continuous glucose monitoring (CGM))
	SD of mean glucose (based on CGM)
	Time in range (TIR) (based on CGM)
	Time in hypoglycaemia (TIH) (based on CGM)
	Total cholesterol
	LDL cholesterol
	HDL cholesterol
	Triglycerides
	P/S-creatinine
	Urine albumin-creatinine ratio
	Albuminuria
	Systolic blood pressure
	Diastolic blood pressure
	Foot screening attendance (date of last visit)
	Eye screening attendance (date of last visit)
	Hypoglycaemia episodes
	Hyperglycaemia emergencies
	Diabetes complications
Patient Reported Outcome	Diabetes self-management behavior performance (DSMQ-R)
	Diabetes-related emotional distress (PAID-5 or DDS2)
	Diabetes-related quality of life (DIDP)
**IBD**	
Case-mix variables – baseline clinical factors	Comorbidities including immune-mediated inflammatory conditions
	Ostomy presence
	Ostomy placement date
	Ostomy removal date
	Vaccination status
Case-mix variables – baseline condition factors	Extra-intestinal manifestations
Case-mix variables – treatment factors	Medication
Patient Reported Outcome	Abdominal pain (PRO-2 CD)
	Stool frequency (liquid or soft) (PRO-2 CD)
	Stool frequency (amount) (PRO-2 UC)
	Rectal bleeding (PRO-2 UC)
	Ostomy symptoms
	Bowel urgency (tenesmus) (Based on Likert Scale)
	Bowel incontinence (daytime) (IBD Disk item ‘Regulating defecation’)
	Bowel incontinence (night-time) (IBD Disk item ‘Regulating defecation’)
	Overall disease control (IBD-Control Item 1a, 5)
	New symptoms (IBD-Control Item 4d)
	Bowel symptoms (IBD-Control Item 2)
	Night bowel symptoms (IBD-Control Item 3b)
	Pain (IBD-Control Item 3c)
	Need for psychological support (IBD-Control Item 3e)
	Every-day life (IBD-Control Item 3a)
	Fatigue (IBD-Control Item 3d)
	Feeling informed
Patient Reported Experience	Therapy satisfaction (IBD-Control Item 3f, 4a, 1b, 4b, 4c)
**LC**	
Clinical outcome	Comorbidities
	Treatment (radiotherapy)
	Treatment (chemotherapy)
	Treatment (immunotherapy)
	Treatment (targeted therapy)
	Treatment (surgery)
Patient Reported Outcome	Health-related quality of life for cancer patients (EORTC-QLQC 30)
	Health-related quality of life (LC13)
**MBC**	
Socio-demographics	Menopausal Status
Clinical outcome	Comorbidities
	Family History
Clinical outcomes – treatment related	Treatment (radiotherapy)
	Treatment (chemotherapy)
	Treatment (immunotherapy)
	Treatment (targeted therapy)
	Treatment (surgery)
Patient Reported Outcome	Health-related quality of life for cancer patients (EORTC-QLQC 30)
	Adverse events: disease symptoms and treatment side effects (PROCTCAE): *Fatigue* *Insomnia* *Shortness of breath/chest tightness* *Pain* *Nausea* *Inflamed and sore mouth* *Hand-foot syndrome*
	Fever (EORTC Item library Q548)

## Appendix C - Composition of Working Groups

**Table:**

Working Group	Responsibility	Participants	Expertise Areas (n)	Role within the H2O Project (n)	Countries (n)	Institution Type
**H2O Taskforce – Core Group**	Curate, analyse, verify, synthesize, gather feedback and formulate implementation steps	10	Academic Researcher (4) PROMs implementation specialist (3) HCP (2) Digital & Data Health Expert (1)	Disease Experts (6) Country Coordinator (4)	Austria (2) Belgium (1) Spain (1) Germany (1) The United Kingdom (1) The Netherlands (3) Switzerland (1)	Public (9) Private (1)
**H2O Taskforce – Validation Group**	Review and validate the proposed model and implementation steps	23	HCPs (7) Academic Researcher (7) Digital & Data Expert (7) Public Health Expert (2)	Country Lead (3) Country Implementing Team (5) Disease Expert (8) Digital Health Expert (3) Technical Expert (4)	Austria (5) Spain (2) The United Kingdom (5) Switzerland (4) The Netherlands (3) Belgium (2) Germany (1) The United States (1)	Public (16) Private (7)
**Country Team – Spain**	Collect, compile, and update local implementation data (for Spain)	12	HCPs (9) Specialists Nurse (1) Digital & Data Health (1) PROMs implementation specialist (1)	Country implementing team (12)	Spain (12)	Public (12)
**Country Team – Germany**	Collect, compile, and update local implementation data (for Germany)	7	HCPs (5) Patient (1) PROMs implementation specialist (1)	Country implementing team (7)	Germany (7)	Public (7)
**Country Team – Austria**	Collect, compile, and update local implementation data (for Austria)	17	HCPs (8) Specialist Nurse (2) Patient (1) Digital & Data Expert (3) Academic researcher (3)	Country implementing team (17)	Austria (17)	Public (17)
**Country Team – The Netherlands**	Collect, compile, and update local implementation data (for The Netherlands)	11	HCPs (4) Digital & Data Health (4) Academic researcher (2) PROMs implementation specialist (1)	Country implementing team (11)	The Netherlands (11)	Public (8) Private (3)

## Appendix D – Identification of barriers and facilitators across the 10 Steps for ePROM implementation

**Table:**

STEP	Barriers	Facilitators
**1. PRO App Collection**	• Different PRO or EHR solutions across practices may increase harmonization complexity • Integration of the app with the EHR can be technically complex and resource-intensive • App must meet local governance standards • Divergent and unique implementation challenges across various sites involved • Digital literacy of patients may be a barrier • Limited engagement or buy-in from clinicians	• Empower each site/country to make decisions and adapt implementation to local workflows. • Assess application technical capabilities and integration into local system • Allow local centres to first begin implementing PROs in disease areas they believe is most feasible (start with one disease at a time) • Establish feedback loops for continuous improvement during rollout
**2. Patient Journey**	• Clinician resistance may occur due to extra time requirements • Patient completion may be limited due to high PRO reporting burden • Implementation of PROMs introduces additional ‘invisible work’ for clinical teams, including patient activation, technical support, monitoring completion, and follow-up coordination • PRO data collection not part of routine practice for most clinical teams/patients • Wide variety of workflow/procedures may exist even within the same practice setting	• Capture PRO data at points of care where patients are typically waiting, or outside of the clinical setting • Focus on capturing what matters most and pragmatic outcome sets first • Patient and clinician involvement in mapping journey • Integrate PROM collection into existing clinical workflows to minimize disruption • Offer reminders and support through automated systems or staff follow-up • Train clinical staff on the importance and use of PROM data to foster engagement
**3. Patient Agreement**	• Patient level of education/understanding of tasks involved varies considerably • Long term patient acceptance can be a challenge • Ethical/Legal requirements may differ based on setting	• Dedicate research staff/clinicians to explain purpose of the PRO initiative to patients • Integrate patient consent/patient agreement into a patient facing application, whenever possible
**4. Data Dictionary**	• Differences in standardized collection practices may exist across participating centres or stakeholder • Patient burden may increase depending on the volume, frequency, and complexity of PROMs selected for data collection • Some clinical data difficult to retrieve from EHR due to plain text/other non-standard format.	• Work with clinical teams to fine tune data capture platform and ensure clinical relevance. • Align on relevant disease categorizations/definitions between clinical and technical teams • Follow best practice guidance (i.e., COSMIN and ICHOM) • Avoid duplication of reporting between patient facing application and clinical EHR record • Develop and maintain a shared data dictionary to ensure consistency in data elements and definitions across teams and systems
**5. Permissions**	• Cost of PROs can be quite high and require financial support • Identifying copyright holder of older PROs may be challenging, particularly on older PROs • Gaining copyright permissions may also prove challenging • Validated translations may be difficult to find or unavailable for less commonly spoken languages.	• When possible and applicable select PROs which are free to use, with translations readily available • Identify local grants which may provide support for costs of PRO tool. • Robust understanding of PRO licencing/translation challenges at the outcome selection phase • Establish flowchart for streamlined management of copyrights, licenses and translations to support implementation aspects
**6. Informatics**	• Limited automation in ePROM delivery can result in missed collection points or added manual workload • Inconsistent use of PROM versions or item phrasing can lead to data quality issues • Technical complexity in configuring calculated fields and ensuring score accuracy across systems. • Clinical system/EHR missing data. • Absence of technological solution to retrieve certain clinical information from EHRs	• Develop comprehensive data dictionaries including outcome categories, item wording, response options, frequency, and scoring logic to standardize data collection across sites • Automate the delivery of ePROMs to ensure timely collection at clinically relevant points in the patient journey • Identify national/internationally implementable and scalable technology solution if possible • Design flexible infrastructures that support both local customization and centralized standards for scaling across contexts • Ensure alignment between clinical and technical teams during configuration
**7. Standardize**	• Lack of early planning regarding where and how PROM data will be stored and accessed within the EHR • Difficulty comparing PROM data across sites due to differences in storage format, structure, or data models • Incompatibility between local data systems and common standards (e.g., OMOP CDM) may complicate harmonization across centres • Lack of clear legal guidance on the reuse and sharing of PROM data across sites and countries • Lack of understanding of relationship between PROs and more traditional clinical outcomes • PRO data collection is not part of hospital management strategies • When a local site has an integrated solution in place, adopting an alternative platform can be challenging even if this may limit broader standardisation across participating centres.	• Engage clinical teams early to determine where and how PROM data will be stored and accessed within the EHR • Adopt shared data standards and formats (e.g., OMOP CDM) to enable comparability of PROM data across sites • Align outcome sets with national initiatives for healthcare professional buy in • Provide training and evidence on how PROMs complement clinical outcomes to support wider clinical acceptance • Ensure integration with hospital management strategies or initiatives (i.e. quality initiatives) • Linking PROs and Clinical outcomes (assess association between these types of assessments)
**8. Interoperability**	• Scaling to private practises may require additional (technological) solutions • Difficulties with technological/data infrastructure which allows for integration and retrieval of clinical data • Lack of clear data flow architecture and planning	• Develop the system architecture and data management plan (DMP) early, in collaboration with local IT departments • Leverage knowledge of internal or external initiatives to align with ongoing efforts • Ensure interoperability with existing registries, which may already be integrating clinical data from the participating centre • Use standardized clinical terminologies and coding systems to enable consistent data mapping across platform
**9. Train**	• Training patients and clinicians on technology/app use may require time and planning • Limited staff availability and lack of time pose challenges for training and implementation • An increased level of oversight is often needed during the initial implementation phase	• Appoint a dedicated person or team to support the clinical team during the initial phase of data collection and implementation • Organise peer-to-peer implementation workshops • Facilitate exchanges between clinicians involved in similar external PRO collection initiatives • Select user-friendly digital solutions that minimise the need for extensive training • Plan patient engagement campaigns • Develop tailored training materials for both patients and healthcare professionals, explaining how to use the app and clear information about the PROMs.
**10. Communicate**	• Clinicians may see the initiative as a single study, rather than part of a broader cultural and systems-level transformation • Clinicians and patients may initially struggle to recognise the value of routine PRO collection	• Develop user-friendly dashboards to visualise PROM results in a clear, accessible way for both patients and clinicians • Ensure PROM data are readily available within clinical workflows to support real-time use and discussion during consultations • Facilitate dedicated time during clinical visits for clinicians to review and discuss ePROM results with patients • Emphasise the potential for PROMs to contribute to broader research and healthcare system improvement • Appoint a liaison role to bridge clinical and technical teams and facilitate communication • Involving heads/clinical champions to support uptake • Patients asking clinicians to participate in PRO collection activities would be encouraging for buy-in

## Abbreviations

COS: Core Outcome Set
COSMIN: COnsensus-based Standards for the selection of health Measurement INstruments
DMP: Data Management Plan
EDHEN: European Health Data & Evidence Network
HER: Electronic Health Record
ePROM: Electronic Patient-Reported Outcome Measures
FHIR: Fast Healthcare Interoperability Resources
H2O: Health Outcomes Observatory
HL7: Health Level 7
IBD: Inflammatory Bowel Disease
ICHOM: International Consortium for Health Outcomes Measurement
IMI: Innovative Medicines Initiative
IT: Information Technology
OMOP CDM: Observational Medical Outcomes Partnership Common Data Model
PAB: Patient Advisory Board
PRO: Patient-Reported Outcome
PROM: Patient-Reported Outcome Measures
PROMIS: Patient-Reported Outcomes Measurement Information System
