# Supplementary material for: A 10-step guide to standardising patient-reported outcomes data collection in healthcare: insights from the health outcomes observatory (H2O) project on overcoming implementation barriers
Source: J Patient Rep Outcomes. 2025 Nov 28;9:138. doi: 10.1186/s41687-025-00958-2 (PMC12662975; doi:10.1186/s41687-025-00958-2)
Supplement: Supplementary file 1 — Supplementary Material 1 [file 41687_2025_958_MOESM1_ESM.docx]

### H2O Pragmatic Core Outcome Set

| **Category** | **Variable** |
| --- | --- |
| **Generic Items** | |
| Socio-demographics | Date of birth |
|  | Sex |
|  | Smoking status |
|  | Alcohol use |
|  | Weight |
|  | Height |
| Case-mix variables – patient-related | Healthcare access |
|  | Disease Diagnosis |
|  | Year of Diagnosis |
| Patient Reported Outcome | Overall well-being (PROMIS -10, 29 or 33 items) |
| **Diabetes Type 1 and Type 2** | |
| Clinical outcome | Pharmacological treatment modality |
|  | Technological treatment modality |
|  | HbA1c |
|  | Mean glucose (based on continuous glucose monitoring (CGM)) |
|  | SD of mean glucose (based on CGM) |
|  | Time in range (TIR) (based on CGM) |
|  | Time in hypoglycaemia (TIH) (based on CGM) |
|  | Total cholesterol |
|  | LDL cholesterol |
|  | HDL cholesterol |
|  | Triglycerides |
|  | P/S-creatinine |
|  | Urine albumin-creatinine ratio |
|  | Albuminuria |
|  | Systolic blood pressure |
|  | Diastolic blood pressure |
|  | Foot screening attendance (date of last visit) |
|  | Eye screening attendance (date of last visit) |
|  | Hypoglycaemia episodes |
|  | Hyperglycaemia emergencies |
|  | Diabetes complications |
| Patient Reported Outcome | Diabetes self-management behavior performance (DSMQ-R) |
|  | Diabetes-related emotional distress (PAID-5 or DDS2) |
|  | Diabetes-related quality of life (DIDP) |
| **IBD** | |
| Case-mix variables – baseline clinical factors | Comorbidities including immune-mediated inflammatory conditions |
|  | Ostomy presence |
|  | Ostomy placement date |
|  | Ostomy removal date |
|  | Vaccination status |
| Case-mix variables – baseline condition factors | Extra-intestinal manifestations |
| Case-mix variables – treatment factors | Medication |
| Patient Reported Outcome | Abdominal pain (PRO-2 CD) |
|  | Stool frequency (liquid or soft) (PRO-2 CD) |
|  | Stool frequency (amount) (PRO-2 UC) |
|  | Rectal bleeding (PRO-2 UC) |
|  | Ostomy symptoms |
|  | Bowel urgency (tenesmus) (Based on Likert Scale) |
|  | Bowel incontinence (daytime) (IBD Disk item ‘Regulating defecation’) |
|  | Bowel incontinence (night-time) (IBD Disk item ‘Regulating defecation’) |
|  | Overall disease control (IBD-Control Item 1a, 5) |
|  | New symptoms (IBD-Control Item 4d) |
|  | Bowel symptoms (IBD-Control Item 2) |
|  | Night bowel symptoms (IBD-Control Item 3b) |
|  | Pain (IBD-Control Item 3c) |
|  | Need for psychological support (IBD-Control Item 3e) |
|  | Every-day life (IBD-Control Item 3a) |
|  | Fatigue (IBD-Control Item 3d) |
|  | Feeling informed |
| Patient Reported Experience | Therapy satisfaction (IBD-Control Item 3f, 4a, 1b, 4b, 4c) |
| **LC** | |
| Clinical outcome | Comorbidities |
|  | Treatment (radiotherapy) |
|  | Treatment (chemotherapy) |
|  | Treatment (immunotherapy) |
|  | Treatment (targeted therapy) |
|  | Treatment (surgery) |
| Patient Reported Outcome | Health-related quality of life for cancer patients (EORTC-QLQC 30) |
|  | Health-related quality of life (LC13) |
| **MBC** | |
| Socio-demographics | Menopausal Status |
| Clinical outcome | Comorbidities |
|  | Family History |
| Clinical outcomes – treatment related | Treatment (radiotherapy) |
|  | Treatment (chemotherapy) |
|  | Treatment (immunotherapy) |
|  | Treatment (targeted therapy) |
|  | Treatment (surgery) |
| Patient Reported Outcome | Health-related quality of life for cancer patients (EORTC-QLQC 30) |
|  | Adverse events: disease symptoms and treatment side effects (PROCTCAE):  *Fatigue*  *Insomnia*  *Shortness of breath/chest tightness*  *Pain*  *Nausea*  *Inflamed and sore mouth*  *Hand-foot syndrome* |
|  | Fever (EORTC Item library Q548) |
